# Retrograde Cricopharyngeal Dysfunction: A Review

**DOI:** 10.3390/jcm13020413

**Published:** 2024-01-11

**Authors:** Mattea E. Miller, Ioan Lina, Lee M. Akst

**Affiliations:** Department of Otolaryngology-Head and Neck Surgery, Johns Hopkins School of Medicine, 601 N Caroline Street, 6th Floor, Suite 6251, Baltimore, MD 21287, USA; mkeiste2@jhmi.edu (M.E.M.);

**Keywords:** retrograde cricopharyngeal dysfunction, inability to burp, review, quality of life, social media

## Abstract

Retrograde cricopharyngeal dysfunction (RCPD), also referred to as retrograde cricopharyngeus dysfunction, is a condition characterized by the inability to burp. The pathophysiology of this condition is thought to result from failure of cricopharyngeal sphincter relaxation during periods of esophageal distension, which leads to patients’ bothersome symptoms. RCPD negatively impacts patients’ quality of life and is associated with bloating, gurgling, avoidance of carbonation, self-imposed dietary and lifestyle changes designed to minimize discomfort, and flatulence. Complaints often start during adolescence, and many patients search for a diagnosis for years before obtaining treatment. A recent increase in awareness through patient-led social media discussion boards describing the ’no burp‘ syndrome is leading to an increasing incidence of presentations, often with patients making a self-diagnosis. The increased incidence of RCPD is fueling a larger case series investigating treatment options and outcomes. In this review, we discuss what is known about the pathophysiology of this condition, the otolaryngologic perspective on diagnosis and treatment, the patients’ lived experience of this condition, and the influence of social media on RCPD.

## 1. Introduction

Retrograde cricopharyngeal dysfunction (RCPD) is a condition that was first described by Kahrilas et al. [1] in 1987 as a dysfunction of the upper esophageal sphincter (UES) causing gurgling noises, pain, and profound esophageal distention. As a result of the symptoms associated with RCPD, several terms were used to describe the condition, including ‘inability to belch’, abelchia, and ‘inability to belch syndrome’. Following a dearth of literature for several decades, a recently published case series of 51 patients by Bastian et al. in 2019 coined the term RCPD [2]. The rebranding of RCPD has subsequently increased awareness amongst physicians and patients and fueled new discussions on pathophysiology, symptoms, and treatment. The diagnosis of RCPD to date is based primarily on symptomatology, specifically (1) an inability to burp; (2) abdominal fullness/bloating; (3) gurgling noises in the chest/lower neck; and (4) excessive flatulence [2,3,4,5,6]. Bastian et al. also reported additional symptoms, including occasional substernal chest pressure and pain. While the absence of concurrent gastrointestinal or oropharyngeal pathology is critical for the diagnosis of RCPD, manometry studies assessing cricopharyngeal muscle function following chemodenervation suggest that impaired relaxation of the cricopharyngeus (CP) contributes to the pathophysiology of RCPD [3]. In this review, we discuss what is known about the pathophysiology of this condition, the otolaryngologic perspective on diagnosis and treatment, the patients’ lived experience of this condition, and the influence of social media on RCPD.

## 2. Pathophysiology

As awareness increases, investigations into the pathophysiology of RCPD are ongoing [2,3,7,8]. What we do know exists largely from case series, retrospective studies, and an understanding of upper esophageal sphincter (UES) physiology and the burping reflex. The symptoms of RCPD are thought to occur due to failure of cricopharyngeal sphincter relaxation under periods of esophageal distension, which prevents normal burping [2,9]. Questions remain regarding the role of functional dysphagia in propagating RCPD symptoms, as patients with RCPD have improved RCPD symptoms long after the effects of botulinum toxin are known to wear off.

### 2.1. The Upper Esophageal Sphincter

The upper esophageal sphincter (UES) is made of the inferior constrictor, the CP, and the proximal esophagus. The function of the UES is to relax during the initial phase of swallowing, prevent regurgitation of esophageal contents, and limit air from entering the stomach during inspiration [10]. Anatomic studies indicate that the CP is the principle contractile component of the upper esophageal sphincter and inserts on the lateral aspects of the cricoid cartilage [9,11,12,13,14]. The CP is largely responsible for generating basal tone in the upper esophageal sphincter, thus preventing reflux into the airway [11,15,16]. The CP is composed of striated muscle and is made up of around 40% elastic connective tissue, much of which is elastic, giving the muscle passive residual tone even in the absence of neural excitation [9]. The UES is innervated via the pharyngeal plexus, supplied by multiple branches of the vagus nerve, glossopharyngeal nerve, and sympathetics via the cranial cervical ganglion, allowing the UES to participate in an array of reflexes [9]. Unlike other sphincters that have a constant tone generated by neural input or intrinsic contractility of the muscle, the UES does not exhibit a constant neurogenic or myogenic tone, as demonstrated by cricopharyngeal electromyography in animal studies and in humans undergoing prolonged UES recordings [9,10,17]. UES contraction and relaxation (though to a lesser degree) are under partial volitional control, and voluntary relaxation is critical during the burping reflex [9,18]. Unlike in other etiologies of cricopharyngeal dysfunction, patients with RCPD do not endorse dysphagia; the syndrome is defined by an inability to burp in the absence of dysphagia.

### 2.2. Burping Reflex

The burping reflex occurs as a sequence of discrete steps: lower esophageal sphincter relaxation, gas reflux from the stomach into the esophagus leading to esophageal distension, UES relaxation, and esophago-pharyngeal gas reflux to restore baseline intra-esophageal pressure [19]. High-resolution esophageal manometry (HRM) and impedance measurements show that transient lower esophageal sphincter (LES) relaxation allows the esophagus to accommodate an air bolus. In patients who can burp, this esophageal distention, which results from gas entering the esophagus from the stomach, leads to UES relaxation, allowing air to release [9]. The relationship between lower esophageal sphincter (LES) relaxation and gastric venting has previously been studied using HRM. In a study of normal volunteers who underwent HRM, Pandolfino et al. observed that during a 2-h postprandial period, over 200 LES relaxation events were observed, and 79% of these were associated with brief periods of UES relaxation, most commonly after gas reflux, as evident [20]. Pandolfino et al. additionally found that the UES relaxations were often short and not associated with audible burps, leading them to coin the term ’microburps‘ [20]. In some instances, UES relaxation occurred in anticipation of gas reflux to the proximal esophagus, which suggests that these normal participants had some volitional control over these microburps [20]. While the exact pathophysiology of RCPD is unknown, a leading hypothesis is that LES relaxation allows for appropriate esophageal distention with retrograde flow of gastroesophageal gas; however, the response of the upper esophageal sphincter (UES) is impaired in RCPD secondary to dysfunctional relaxation [9]. Therefore, the patients cannot belch, and the air remains ‘trapped’ in the esophagus. This creates a substernal pressure sensation until peristalsis ‘swallows’ the air back down into the stomach. The motion of this gas as it is released from the stomach to the esophagus, then swallowed, and then vented again results in the ’gurgling‘ sound described by most patients with RCPD.

### 2.3. The Role of Behavior?

As we learn more about the potential role of UES sphincter relaxation and the burping reflex in RCPD, the role of behavior in propagating RCPD symptoms remains unclear. In a study performed on patients diagnosed with RCPD who were treated with botulinum toxin injections into the CP Nijhuis et al. observed a complete response to botulinum toxin injection in all participants [3]. They suggested that an alteration in neurophysiological function, either motor or sensory, underlies the inability of the UES to relax, rather than an ineffective stimulus [3]. HRM performed prior to botulinum toxin injection in a series of patients with RCPD revealed a paradoxical increase in UES pressure followed by secondary peristalsis, suggesting that in patients with RCPD, the inability for the UES to relax in response to esophageal distention may be a subconsciously learned behavioral response to avoid aspiration [3]. Further research is warranted to evaluate the role of behavior in RCPD.

## 3. Diagnosis

The diagnosis of RCPD is a diagnosis of exclusion and relies primarily on clinical history [2,5,9]. As a result, the diagnostic workup is variable and typically unremarkable [3]. Many published case series have not identified a definitive diagnostic test (esophagoscopy, modified barium swallow, esophagram, or esophageal manometry) that reliably diagnoses RCPD [7]. However, many of these studies have focused on the use of diagnostic testing, excluding other pharyngeal and esophageal pathologies [21]. Recent work performed by Oudi Nijhius et al. highlights high-resolution manometry findings in patients with RCPD after a carbonated-drink provocation test, which may be useful for diagnosing patients with RCPD in the future [3]. To date, the ability of cricopharyngeal muscle botulinum toxin injections to restore burping has been considered both diagnostic of RCPD and therapeutic [2,5,7,9,22,23].

## 4. Lived Experience of RCPD

RCPD significantly impacts patients’ quality-of-life. People living with RCPD often report never being able to burp with bothersome symptoms starting in adolescence that continue to worsen into adulthood [5]. Qualitative interviews conducted with patients living with RCPD revealed that patients with RCPD are most affected by symptoms of bloating and gurgling noises, which cause increased anxiety, social isolation, and decreased productivity (days absent from work and/or school) [24]. In search of reprieve, patients make lifestyle modifications, change their exercise regimen, limit or modify their diet, and try remedies posted on online forums such as ‘air vomiting’ (self-inducing vomiting to release trapped air in the stomach) in efforts to minimize their symptoms [24].

## 5. Treatment

Treatment of RCPD is variable and can be performed either in the office or in the operating room. Typically, treatment involves chemodenervation with a botulinum toxin injection of the CP [23]. Other treatments have been described and include balloon dilation of the CP or cricopharyngeal myotomy, either as stand-alone treatments or in conjunction with botulinum toxin injections of the CP [2,7,25,26].

### 5.1. Botulinum Toxin Injections into the Cricopharyngeus Muscle

Botulinum toxin injections into the CP are the mainstay of RCPD treatment. Currently, there is no uniform dosing protocol, and therefore, treating physicians have their own preferences for injection technique and dosing [8,22,23,27]. A study on the long-term efficacy of the first 200 patients with RCPD treated with botulinum toxin injections revealed that 95% of patients experienced relief of the cardinal symptoms of RCPD, and about 80% of those patients had durable resolution in their symptoms at 6 months post-treatment [22]. For patients with recurrent symptoms, repeat botulinum toxin injections often result in subsequent relief of their symptoms [8,22,27]. Although the majority of patients require only a single dose of botulinum toxin, despite its temporary effect, the procedure is not without risk. The most common side effect for patients undergoing CP chemodenervation is transient dysphagia lasting 1–4 weeks following botulinum toxin injection [3,4,8,22,23]. Given the proximity of the posterior cricoarytenoid muscle (PCA) to the CP muscle, there is also a risk of vocal fold paresis secondary to the diffusion of the botulinum toxin in a small number of patients [27]. To this date, it is unclear why the majority of patients maintain the ability to burp indefinitely following the 3-month window of botulinum toxin activity, which suggests that further studies are needed to understand the mechanism. Case series published by Bastian et al. highlight that the therapeutic benefit of botulinum toxin injections for RCPD patients is longer-lasting than the known pharmacological effect of botulinum toxin [2,7,22]. One hypothesis described by Hoesli et al. is that transient weakness of the CP muscle following injection of botulinum toxin allows for patients to retrain CP relaxation and ‘learn’ the burp reflex that had previously been absent [22].

At most institutions, patients with RCPD undergo a CP botulinum toxin injection in the operating room (OR). Direct laryngoscopy and esophagoscopy allow for increased accuracy of injection and the diagnosis of potential associated esophageal pathology [8]. Reported success rates vary from 88.2–95% with a preferred dose of botulinum toxin, which varies from 25U–100U [7,8,22,23,27]. Recently, a case report performed by Pavesi et al. discussed a patient with RCPD who was treated with 10U of botulinum toxin with a durable response at 4 months of clinical follow-up [28]. No studies have identified particular patient characteristics that are associated with the success of initial treatment with botulinum toxin, and further, it is not known what the standard dose of botulinum toxin should be for treatment of RCPD. To date, there has not been a prospective trial evaluating the dose of botulinum toxin needed to treat RCPD, leading to a wide range of doses used across the field. Further research is needed to determine a recommended dosing for patients with RCPD, as treatment with botulinum toxin can result in adverse side effects such as transient dysphagia, reflux, and ineffective injection [8,22,27]. The amount of botulinum toxin utilized has not been associated with the incidence of adverse side effects [8,22].

Recently, several centers have introduced injections of botulinum toxin for RCPD treatment in their offices. In-office treatment offers several advantages over treatment in the OR in that patients can avoid general anesthesia and can be scheduled faster for an in-office appointment. Electromyography is useful in localizing the CP in the office [23,27]. Descriptions of percutaneous access to the CP muscle predate the treatment of RCPD and were used for monitoring the CP and for the treatment of CP hypertonicity [29,30,31,32,33]. A retrospective review of the efficacy and safety of EMG-guided botulinum toxin injections reported that all patients treated by this modality had resolution of their RCPD symptoms [23]. Recently, Doruk and Pitman described using a transcervical lateral approach with an EMG-guided needle to inject botulinum toxin in the left side of CP muscle compared to OR exposure using a diverticular scope and bilateral CP muscle injection [27]. They overall reported a significantly higher success rate of OR injections (90.2%) compared to those performed in the office (64.9%) with similar side effect rates [27].

### 5.2. Cricopharyngeal Dilation

Some centers report using balloon catheter dilation of the UES prior to injection of botulinum toxin into the CP muscle [34,35]. Balloon dilation is most often used to treat CP bar hypertonicity; however, there are reports of this being used for the treatment of RCPD [36]. To date, there have not been large case series reporting on the efficacy of balloon dilation in conjunction with botulinum toxin injections, as many centers forgo dilation.

### 5.3. Myotomy

A case report of cricopharyngeal myotomy used for treatment of RCPD was recently published by Bastian et al. in 2020 [7]. They describe a patient who initially underwent injection of 50U botulinum toxin into the CP with resolution of his RCPD symptoms that ultimately recurred after 5 months. He was retreated with 75U, again with initial resolution and an eventual relapse of his symptoms. In this setting, the treating team offered endoscopic cricopharyngeal myotomy with 80% division of the CP using a CO_2_ laser [7]. They reported no adverse effects of this treatment, and the patient continues to have resolution of their RCPD symptoms at the time of publication [7].

### 5.4. The Role of Speech–Language Pathologists

Speech–Language Pathologists (SLP) often identify and treat patients with swallowing disorders, such as patients with CP dysfunction, and play a crucial role in treating patients with functional disorders [37]. Recently, a study published by Goldstein et al. evaluated the effectiveness of speech language therapy in treating patients with functional speech disorder [38]. In this study, patients attended an average of 9.2 visits over 4.4 months, and at the last treatment session, the majority of these patients had improved in their symptoms, with three individuals becoming completely asymptomatic, providing support for speech language therapy in the management of functional disorders [38]. For patients with RCPD, the role of behavior in propagating RCPD symptoms has not been fully elucidated and warrants further investigation. A case report was recently published that described the resolution of RCPD symptoms following hypnosis therapy, emphasizing the importance of therapy in the treatment of patients with RCPD [39]. A formalized therapy plan to treat patients with RCPD has not yet been developed, though future studies should seek to develop and evaluate a therapy plan for patients with RCPD. In the future, swallowing therapy with a SLP may play a role in the care of patients with RCPD.

## 6. The Role of Social Media

Awareness of RCPD continues to rise since its redescription in 2019, as evidenced by the increased number of case series that have been published on the subject [2,4,5,8,40]. The use of social media by the general public offers a space for patients who are experiencing symptoms of RCPD to connect and share their experiences and resources [41]. Similar to other rare pathologies, patients are now turning to social media platforms such as Reddit^®^, Facebook^®^, TikTok^®^, and YouTube^®^ for medical information [8,41,42]. While social media provides a novel opportunity for community and information sharing, challenges exist when sharing medical information online [43]. Monitoring the accuracy of information shared remains a significant hurdle that is yet to be solved [44,45,46,47,48,49]. The RCPD online community has a significant following, with the Reddit^®^ page ‘r/noburp’ reporting over 22,000 subscribers and close to 5000 new members in the last year alone [42]. Members sharing their symptoms and experiences living with RCPD provide detailed accounts of how to ‘air vomit’ (self-induced vomiting with the goal of allowing trapped air in the stomach out), their diagnosis of RCPD, and their experiences with botulinum toxin treatment. Participants report bringing information they found online to their physicians to facilitate a medical diagnosis of RCPD [24]. Referrals to specific physicians treating RCPD can also be found via social media, with many otolaryngologists being named on social media sites as providing botulinum toxin injections to treat RCPD [8,24,42].

## 7. Conclusions

RCPD has been described as a constellation of symptoms stemming from a dysfunctional burp reflex. The number of patients diagnosed and treated for RCPD has recently increased, largely due to the rebranding of this condition as RCPD in 2019 as well as an increasing awareness on social media platforms. Research screening tools and prospective clinical studies in RCPD will be essential to developing targeted treatment algorithms and improving patient care.

## 8. Future Directions

**Figure 1:**
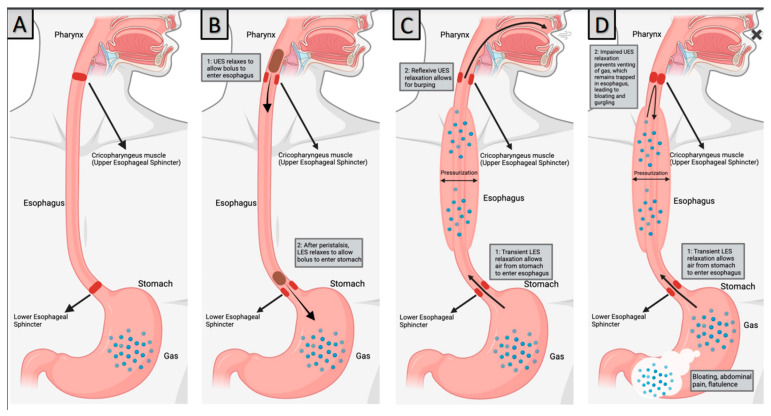
Pathophysiology of RCPD (**A**) Normal anatomy, (**B**) Swallowing, (**C**) Normal Burping, (**D**) RCPD. Created with BioRender.Com.

**Figure 2:**
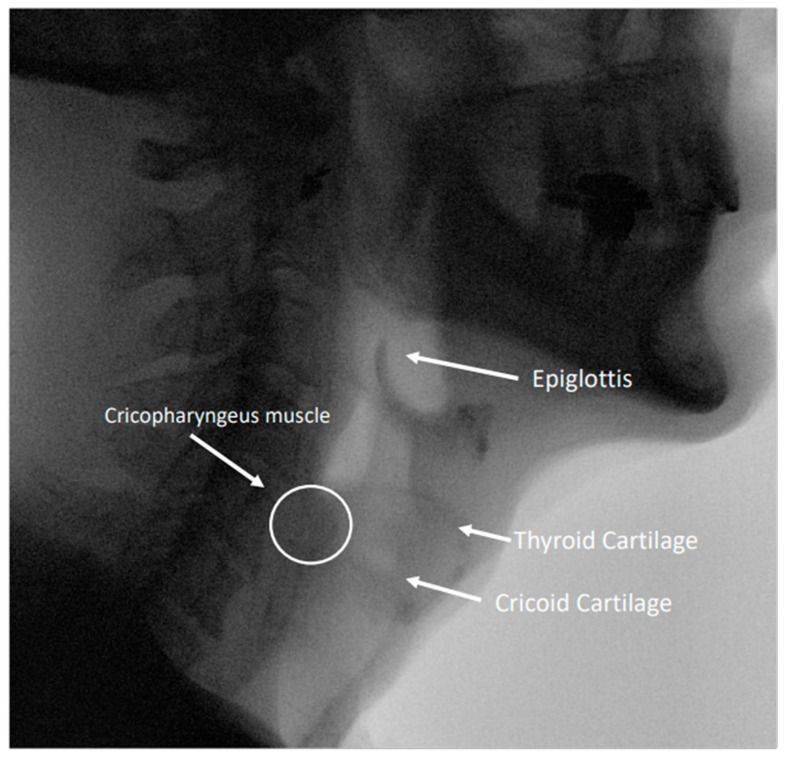
Image from a modified barium swallow study highlighting relevant anatomy in the pharynx and esophagus.

There are many questions that remain to be answered regarding the pathophysiology of RCPD, appropriate diagnostic workup, treatment of this condition, and the role of SLPs in supporting patients with RCPD. The role of behavior in propagating RCPD symptoms should be further examined to date. No studies have explained the mechanism behind symptom resolution that extends beyond the known physiologic mechanism of action for botulinum toxin in a large proportion of patients with RCPD. Additionally, prospective trials evaluating the effective dose of botulinum toxin should be performed with the goal of determining the minimum effective dose for the treatment of RCPD. Future studies on interventions for patients with RCPD should include collaboration with SLPs to evaluate whether a tailored swallowing therapy program would be beneficial for patients with RCPD (Figure 1 and Figure 2).
